# Novel sulphonamides incorporating triazene moieties show powerful carbonic anhydrase I and II inhibitory properties

**DOI:** 10.1080/14756366.2019.1700240

**Published:** 2019-12-09

**Authors:** Sinan Bilginer, Baris Gonder, Halise Inci Gul, Ruya Kaya, Ilhami Gulcin, Baris Anil, Claudiu T. Supuran

**Affiliations:** a Department of Pharmaceutical Chemistry, Faculty of Pharmacy, Ataturk University, Erzurum, Turkey; b Department of Chemistry, Faculty of Science, Ataturk University, Erzurum, Turkey; c Central Research & Application Laboratory, Agri Ibrahim Cecen University, Agri, Turkey; d Neurofarba Department, University Firenze, Florence, Italy

**Keywords:** Carbonic anhydrase, diazonium salt, inhibitors, metanilamide, sulphonamide, triazene

## Abstract

A series of compounds incorporating 3-(3-(2/3/4-substituted phenyl)triaz-1-en-1-yl) benzenesulfonamide moieties were synthesised and their chemical structure was confirmed by physico-chemical methods. Carbonic anhydrase (CA, EC 4.2.1.1) inhibitory effects of the compounds were evaluated against human isoforms hCA I and II. K_I_ values of these sulphonamides were in the range of 21 ± 4–72 ± 2 nM towards hCA I and in the range of 16 ± 6–40 ± 2 nM against hCA II. The 4-fluoro substituted derivative might be considered as an interesting lead due to its effective inhibitory action against both hCA I and hCA II (K_I_s of 21 nM), a profile rarely seen among other sulphonamide CA inhibitors, making it of interest in systems where the activity of the two cytosolic isoforms is dysregulated.

## 1. Introduction

**Figure 1.**
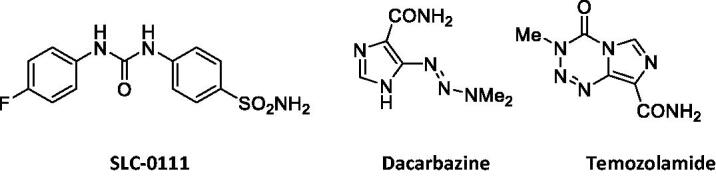
Chemical structures of SLC-0111, Dacarbazine, and Temozolamide.

Carbonic anhydrases (CAs, EC 4.2.1.1) are a superfamily of metalloenzymes that catalyse the interconversion reaction between carbon dioxide and bicarbonate^1^^,^^2^. CAs catalyse this reaction by using a metal hydroxide nucleophilic mechanism^3–5^. The metal ions present within the active site of these enzymes (Zn(II), Cd(II), Co(II), Fe(II) or Mn(II) for the ι-CAs) are coordinated by three amino acid residues and a water molecule which is activated by the metal ion and the hydrophobic environment, becoming highly nucleophilic^3–5^. This metal hydroxide species nucleophilically attacks CO_2_ and promotes its hydration to bicarbonate very efficiently at almost a neutral pH^3–5^. There are eight genetically distinct CA families, namely α-, β-, γ-, δ-, ζ- η-, θ-, and ι-CAs^3^^,^^4^^,^^6^^,^^7^. In humans, there are 12 catalytically active α-CA isoforms which have different catalytic activities^6^. These enzymes are distributed in many organs and tissues and they play important roles in many physiological and pathological processes such as acid-base regulation, biosynthetic reactions, electrolyte secretion, and calcification. Thus, human carbonic anhydrases (hCAs) are targets for the design of new drugs to use in the diagnosis and/or treatment of many diseases. For example, hCA II, IV, XII, and XIV inhibitors are used as diuretics, whereas hCA II, IV, and XII inhibitors are used as anti-glaucoma drugs^1^^,^^2^^,^^8^^,^^9^. hCA I plays a role in the regulation of retinal pathologic processes, and its inhibition is a strategy for the treatment of such conditions. hCA II inhibitors are used for the treatment of glaucoma, oedema, and epilepsy^5^^,^^8^. The cytosolic, highly abundant isoforms hCA I and II are in fact both drug targets for a multitude of diseases, as highlighted above, but due to their role in pH homeostasis and wide distribution in many tissues, are also frequently off-targets, when other isoforms (e.g. CA VA/B, VII, IX or XII) should be selectively inhibited^7^^,^^9^. Although CA inhibitors (CAIs) have been often used in clinics, the first and second-generation drugs have several undesired side effects because of their low selectivities for various isozymes with medicinal chemistry applications^5^^,^^8^^,^^10^^,^^11^. Therefore, there is a need for compounds with higher selectivity to diverse hCAs compared to the available drugs used clinically nowadays. Compounds containing sulphonamide group (R-SO_2_NH_2_) and its isosteres sulfamide (R-NH-SO_2_NH_2_) and sulfamate (R-OSO_2_NH_2_) are among the most important classes of CAIs^12^. Some of these derivatives have been used as drugs for many years^13^. More recently, a compound bearing an ureido-substituted aryl-sulphonamide (SLC-0111) (Figure 1) was reported to show remarkable CA inhibitory effects and is presently in Phase Ib/II clinical trials as an antitumor/antimetastatic agent^14^^,^^15^.

Triazenes are an interesting group of compounds which has many applications in the synthesis of various products, some of which possess pharmacological applications^16^^,^^17^. Dacarbazine and temozolomide (Figure 1), both incorporating a triazene moiety, are in the clinical use for cancer treatment, as they possess acceptable toxicity and good pharmacokinetic properties^17^^,^^18^. Additionally, the triazene group is an isostere of the ureido group and was recently reported to lead to interesting CA inhibitory derivatives by Akocak et al.^19^.

Some compounds bearing triazene substituted sulphanilamide or metanilamide (3-aminobenzene sulphonamide) were recently reported to possess CA inhibitory effects by one of our groups^13^^,^^20^, but there are few studies on this class of CA inhibitors. In this study, we synthesised new 1,3-diaryltriazene sulphonamide compounds^1–12^ from the diazonium salt of metanilamide and different substituted aromatic amines and evaluated their inhibition profiles towards hCA I and II, in order to find out new drug candidates.

## 2. Experimental

### 2.1. Chemistry

All chemicals and solvents were purchased from Sigma-Aldrich and Merck. The nuclear magnetic resonance (NMR) spectra (^1^H NMR, ^13 ^C NMR) were recorded on a Bruker AVANCE III 400 MHz (Bruker, Karlsruhe, Germany) spectrometer [400 MHz (^1^H) and 100 MHz (^13 ^C)] in DMSO-d_6_. Chemical shifts are given as δ values in ppm. Tetramethylsilane was used as the internal standard and *J* values were expressed in Hz. Mass spectra of the compounds were recorded using a liquid chromatography ion trap-time of flight tandem mass spectrometer (Shimadzu, Kyoto, Japan) equipped with an electrospray ionisation (ESI) source, operating in both positive and negative ionisation mode. Shimadzu’s LCMS Solution software was used for data analysis. Melting points were determined using an Electrothermal 9100 instrument (IA9100, Bibby Scientific Limited, Staffordshire, UK) and are uncorrected. Reactions were monitored by Thin Layer Chromatography (TLC) [Silicagel 60 HF254 (Merck KGaA)].

#### 2.1.1. Synthesis of 1,3-diaryltriazene sulphonamide derivatives

To the solution of 3-aminobenzene sulphonamide (5 mmol) in water (3 ml), concanrate HCl (1.5 ml) was added then the mixture was cooled to 0–5 °C and stirred for 5 min. To this mixture, sodium nitrite (7 mmol) in water (3 ml) was added dropwise during about 10–15 min at 0–5 °C. This mixture was stirred about 15–20 min at 0–5 °C. Then, this mixture (diazonium solution) was added to a suitable aniline (5 mmol) solution (in 5 ml methanol) by adjusting the pH between 6 and 7 with the simultaneous addition of saturated sodium acetate in water. The reaction mixture was stirred at 0–5 °C for 3 h and then overnight at room temperature in dark^13^. The precipitated solid product was collected by filtration and washed several times with cold water. The crude compounds were air-dried then purified by crystallization from methanol. The chemical structures of the compounds^1–12^ were characterised by ^1^H NMR, ^13 ^C NMR, and HRMS.

##### 2.1.1.1. 3–(3-Pheynltriaz-1-en-1-yl)benzenesulfonamide (1)

Yield 72.3%. Mp: 146–147 °C. ^1^H NMR (DMSO-d_6_) δ (ppm) 12.71 (s, NH, 1H), 8.2 (s, 1H, Ar-H), 7.98-7.85 (m, 2H, Ar-H), 7.70 (t, 1H, *J* = 7.7 Hz, Ar-H), 7.58-7.50 (m, 2H, Ar-H), 7.49 (s, 2H, –SO_2_NH_2_), 7.20-6.99 (m, 1H, Ar-H), 6.71 (d, 1H, *J* = 7.6 Hz, Ar-H), 6.57 (d, 1H, *J* = 7.6 Hz, Ar-H). ^13 ^C NMR (DMSO-d_6_) δ (ppm) 154.1, 145.8, 143.1, 130.5, 129.8, 129.3, 126.2, 117.8, 114.5, 113.9. HRMS (ESI-MS) *m/z* calculated [M + H]^+^ 277.0681; measured 277.06718.

##### 2.1.1.2. 3-(3-(4-Fluorophenyl)triaz-1-en-1-yl) benzenesulfonamide (2)

Yield 47.2%. Mp: 162–163 °C. ^1^H NMR (DMSO-d_6_) δ (ppm) 12.75 (s, NH, 1H), 7.80 (s, 1H, Ar-H), 7.74-7.68 (m, 1H, Ar-H), 7.65-7.59 (m, 1H, Ar-H), 7.56-7.44 (m, 3H, Ar-H), 7.43 (s, 2H, –SO_2_NH_2_), 7.32-6.24 (m, 2H, Ar-H). ^13 ^C NMR (DMSO-d_6_) δ (ppm) 146.2, 145.4, 130.0, 122.7, 119.1, 117.2, 116.2, 115.9, 110.85, 110.83. HRMS (ESI-MS) *m/z* calculated [M + H]^+^ 295.05867; measured 295.05786.

##### 2.1.1.3. 3-(3-(4-Bromophenyl)triaz-1-en-1-yl) benzenesulfonamide (3)

Yield 53.0%. Mp: 159–160 °C. ^1^H NMR (DMSO-d_6_) δ (ppm) 7.89 (d, 1H, *J* = 1.86 Hz, Ar-H), 7.60-7.59 (m, 1H, Ar-H), 7.58 (d, 1H, *J* = 7.0 Hz, Ar-H), 7.56-7.55 (m, 3H, Ar-H), 7.54 (s, 2H, –SO_2_NH_2_), 7.42 (dd, 1H, *J_1_* = 7.0 Hz, *J_2_*= 1.86 Hz, Ar-H), 7.38-7.36 (m, 1H, Ar-H). ^13 ^C NMR (DMSO-d_6_) δ (ppm) 174.7, 146.5, 145.8, 134.2, 132.6, 130.5, 121.4, 120.1, 117.6, 114.4. HRMS (ESI-MS) *m/z* calculated [M + H]^+^ 354.97861; measured 354.97882.

##### 2.1.1.4. 3-(3-(4-Ethoxyphenyl)triaz-1-en-1-yl) benzenesulfonamide (4)

Yield 42.7%. Mp: 152–154 °C. ^1^H NMR (DMSO-d_6_) δ (ppm) 12.45 (s, NH, 1H), 7.79 (s, 1H, Ar-H), 7.52 (d, 2H, *J* = 7.8 Hz, Ar-H), 7.45-7.43 (m, 3H, Ar-H), 7.00 (d, 2H, *J* = 7.8 Hz, Ar-H), 4.07-4.05 (m, 2H, –CH_2_–), 1.35 (t, 3H, *J* = 6.5 Hz, –CH_3_).^13^C NMR (DMSO-d_6_) δ (ppm) 158.6, 145.8, 143.4, 142.9, 130.5, 122.7, 119.0, 117.2, 115.4, 110.9, 63.8, 15.1. HRMS (ESI-MS) *m/z* calculated [M + H]^+^ 321.09431; measured 321.09354.

##### 2.1.1.5. 3-(3-(4-Methoxyphenyl)triaz-1-en-1-yl) benzenesulfonamide (5)

Yield 40.0%. Mp: 133–134 °C. ^1^H NMR (DMSO-d_6_) δ (ppm) 12.46 (s, NH, 1H), 7.80 (s, 1H, Ar-H), 7.54 (d, 2H, *J* = 8.2 Hz, Ar-H), 7.53-7.43 (m, 3H, Ar-H), 7.42 (s, 2H, –SO_2_NH_2_), 7.02 (d, 2H, *J* = 8.2 Hz), 3.80 (s, 3H, –OCH_3_). ^13 ^C NMR (DMSO-d_6_) δ (ppm) 159.3, 145.8, 143.6, 142.9, 130.5, 122.7, 119.0, 117.2, 114.9, 110.9, 55.8. HRMS (ESI-MS) *m/z* calculated [M + H]^+^ 307.07866; measured 307.07784.

##### 2.1.1.6. 3-(3-(4-Ethylphenyl)triaz-1-en-1-yl) benzenesulfonamide (6)

Yield 19.3%. Mp: 151 °C. ^1^H NMR (DMSO-d_6_) δ (ppm) 12.66 (s, NH, 1H), 7.92 (s, 1H, Ar-H), 7.66 (d, 1H, *J* = 4.8 Hz, Ar-H), 7.59 (d, 1H, *J* = 7.7 Hz, Ar-H), 7.52-7.41 (m, 3H, Ar-H), 7.40 (s, 2H, -SO_2_NH_2_), 7.27 (d, 1H, *J* = 7.4 Hz, Ar-H), 7.19 (d, 1H, *J* = 7.7 Hz, Ar-H), 2.59 (q, 2H, *J* = 7.5 Hz, –CH_2_), 1.19 (t, 3H, J = 7.5 Hz, –CH_3_).^13^C NMR (DMSO-d_6_) δ (ppm) 148.1, 145.9, 143.5, 130.7, 129.3, 124.9, 121.6, 119.5, 117.6, 111.2, 28.5, 16.4. HRMS (ESI-MS) *m/z* calculated [M + H]^+^ 305.0994; measured 305.0985.

##### 2.1.1.7. 3-(3-(3-Chlorophenyl)triaz-1-en-1-yl) benzenesulfonamide (7)

Yield 63.3%. Mp: 136–137 °C. ^1^H NMR (DMSO-d_6_) δ (ppm) 12.86 (d, NH, 1H, *J* = 23.1 Hz), 8.17 (d, 1H, *J* = 1.7 Hz, Ar-H), 8.00-7.83 (m, 1H, Ar-H), 7.80-7.70 (m, 1H, Ar-H), 7.62-7.47 (m, 2H, Ar-H), 7.49 (s, 2H, –SO_2_NH_2_), 6.80 (d, 1H, *J* = 2.3 Hz, Ar-H), 6.62 (dd, 1H, *J_1_* = 8.9 Hz, *J_2_*= 2.3 Hz, Ar-H), 6.57 (s, 1H, Ar-H).^13^C NMR (DMSO-d_6_) δ (ppm) 154.8, 145.8, 134.4, 131.5, 130.6, 127.4, 124.4, 122.8, 120.7, 117.9, 113.5, 111.5. HRMS (ESI-MS) *m/z* calculated [M + H]^+^ 311.02912; measured 311.02855.

##### 2.1.1.8. 3-(3-(3-Fluorophenyl)triaz-1-en-1-yl) benzenesulfonamide (8)

Yield 35.7%. Mp: 154–156 °C. ^1^H NMR (DMSO-d_6_) δ (ppm) 12.87 (d, NH, 1H, *J* = 16.7 Hz), 7.99 (s, 1H, Ar-H), 7.84 (s, 1H, Ar-H), 7.78-7.73 (m, 1H, Ar-H), 7.66-7.62 (m, 1H, Ar-H), 7.54-7.41 (m, 2H, Ar-H), 7.21-7.13 (m, 1H, Ar-H), 6.90-6.86 (m, 1H, Ar-H). ^13 ^C NMR (DMSO-d_6_) δ (ppm) 151.9, 150.4, 145.7, 131.6, 130.5, 125.1, 124.4, 120.0, 118.4, 117.8, 111.5, 111.0. HRMS (ESI-MS) *m/z* calculated [M + H]^+^ 295.05867; measured 295.05832.

##### 2.1.1.9. 3-(3-(3-Methoxyphenyl)triaz-1-en-1-yl) benzenesulfonamide (9)

Yield 43%. Mp: 201 °C. ^1^H NMR (DMSO-d_6_) δ (ppm) 8.11 (s, 1H, Ar-H), 7.92 (d, 1H, *J* = 7.6 Hz, Ar-H), 7.82 (d, 1H, *J* = 7.2 Hz, Ar-H), 7.71-7.54 (m, 2H, Ar-H), 7.49 (s, 2H, –SO_2_NH_2_), 6.40-6.26 (m, 3H, Ar-H), 3.89 (s, 3H, –OCH_3_). ^13 ^C NMR (DMSO-d_6_) δ (ppm) 160.1, 155.6, 153.0, 145.1, 132.5, 129.9, 126.1, 125.2, 117.9, 117.1, 106.9, 95.9, 55.4. HRMS (ESI-MS) *m/z* calculated [M + H]^+^ 307.07866; measured 307.07828.

##### 2.1.1.10. 3-(3-(2-Chlorophenyl)triaz-1-en-1-yl) benzenesulfonamide (10)

Yield 48.4%. Mp: 149–150 °C. ^1^H NMR (DMSO-d_6_) δ (ppm) 13.12 (s, NH, 1H), 7.89 (s, 1H, Ar-H), 7.68 (d, 1H, *J* = 8.0 Hz, Ar-H), 7.58-7.42 (m, 3H, Ar-H), 7.46 (s, 2H, –SO_2_NH_2_), 7.45-7.39 (m, 2H, Ar-H), 7.34-7.27 (m, 1H, Ar-H). ^13 ^C NMR (DMSO-d_6_) δ (ppm) 145.9, 142.5, 130.74, 130.73, 130.6, 129.4, 128.8, 128.4, 120.2, 119.7, 118.0, 111.6. HRMS (ESI-MS) *m/z* calculated [M + H]^+^ 311.02912; measured 311.02941.

##### 2.1.1.11. 3-(3-(2-Fluorophenyl)triaz-1-en-1-yl) benzenesulfonamide (11)

Yield 34.2%. Mp: 167–168 °C. ^1^H NMR (DMSO-d_6_) δ (ppm) 13.06 (s, NH, 1H), 7.82 (s, 1H, Ar-H), 7.72-7.67 (m, 1H, Ar-H), 7.56-7.46 (m, 3H, Ar-H), 7.43 (s, 2H, –SO_2_NH_2_), 7.33-7.31 (m, 2H, Ar-H), 7.28-7.25 (m, 1H, Ar-H). ^13 ^C NMR (DMSO-d_6_) δ (ppm) 145.9, 142.3, 130.7, 129.1, 129.0, 125.4, 120.0, 119.7, 117.8, 117.3, 117.1, 111.4. HRMS (ESI-MS) *m/z* calculated [M + H]^+^ 295.05867; measured 295.05799.

##### 2.1.1.12. 3-(3-(2-Bromophenyl)triaz-1-en-1-yl) benzenesulfonamide (12)

Yield 13%. Mp: 157 °C. ^1^H NMR (DMSO-d_6_) δ (ppm) 13.08 (s, NH, 1H), 7.87 (s, 1H, Ar-H), 7.72 (d, 1H, *J* = 7.9 Hz, Ar-H), 7.63 (d, 1H, *J* = 7.9 Hz, Ar-H), 7.54-7.45 (m, 4H, Ar-H), 7.44 (s, 2H, –SO_2_NH_2_), 7.25 (d, 1H, *J* = 7.4 Hz, Ar-H). ^13 ^C NMR (DMSO-d_6_) δ (ppm) 147.7, 145.9, 142.3, 133.8, 130.6, 129.2, 129.0, 120.2, 119.9, 119.8, 118.0, 111.6. HRMS (ESI-MS) *m/z* calculated [M + H]^+^ 354.97861; measured 354.97882.

### 2.2. Carbonic anhydrase inhibition assay

CA inhibition assay was done as described in our previous studies by using an esterase assay with 4- nitrophenyl acetate as standard^21–31^. The enzymes were purified from human blood as described earlier^27^^,^^28^.

## 3. Discussion

### 3.1. Chemistry

**Scheme 1.**
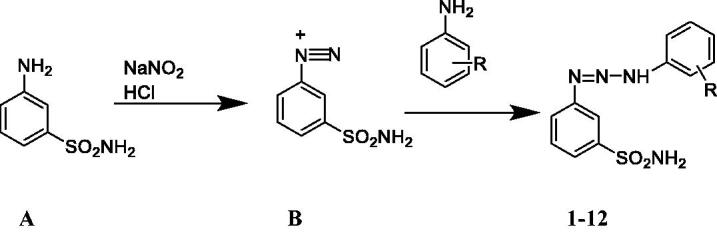
General synthetic pathway for compounds **1–12**.

Compounds **1–12**, 3-(3-(2/3/4-substituted phenyl)triaz-1-en-1-yl) benzenesulfonamide, were synthesised and purified successfully for the first time (except **2** and **5** reported eralier^13^) as shown in Scheme 1. The diazonium salt obtained from the 3-aminobenzenesulfonamide **A** was reacted with sodium nitrite (in the presence of a strong acid) generating the diazonium salts **B**, which were treated with the suitable aniline derivative, leading to triazenes **1–12**. The anilines used were: unsubstituted aniline (**1**), 4-fluoroaniline (**2**), 4-bromoaniline (**3**), 4-ethoxyaniline (**4**), 4-methoxyaniline (**5**), 4-ethylaniline (**6**), 3-chloroaniline (**7**), 3-fluoroaniline (**8**), 3-methoxyaniline (**9**), 2-chloroaniline (**10**), 2-fluoroaniline (**11**), and 2-bromoaniline (**12**) in the series. Compound **1**, the non-substituted derivative, was synthesised with the highest yield (% 72.3) whereas the 2-bromo substituted derivative compound **12** was synthesised with the lowest yield (% 13) in the series.

The chemical structures of the compounds **1–12** were characterised by ^1^H NMR, ^13 ^C NMR, and HRMS (see the experimental part for details).

### 3.2. Carbonic anhydrase inhibitory effects

CA inhibition with compounds **1–12** on hCA I and hCA II are shown in Table 1. Acetazolamide (**AZA**) was used as a reference drug. According to Table 1, K_I_ values (inhibitory potency) of the compounds **1–12** were in the range of 21 ± 4–72 ± 2 nM towards hCA I while they were in the range of 16 ± 6–40 ± 2 nM towards hCA II. K_I_ values of **AZA** were 19 ± 2 nM and 17 ± 4 nM towards hCA I and hCA II, respectively (by an esterase method, using 4-nitrophenyl acetate as substrate).

**Table 1.** Inhibitory effects of the compounds **1–12** on hCA I and II isoenzymes by an esterase, 4-nitrophenyl acetate assay.


		K_I_(nM)	
Compounds	R	hCA I	hCA II
**1**	H	28 ± 6	18 ± 5
**2**	4-F	21 ± 4	21 ± 9
**3**	4-Br	57 ± 3	19 ± 4
**4**	4-EtO	38 ± 4	16 ± 6
**5**	4-MeO	42 ± 8	21 ± 4
**6**	4-Et	49 ± 2	16 ± 2
**7**	3-Cl	53 ± 15	22 ± 6
**8**	3-F	36 ± 3	28 ± 6
**9**	3-MeO	33 ± 5	23 ± 2
**10**	2-Cl	46 ± 12	20 ± 5
**11**	2-F	72 ± 2	33 ± 9
**12**	2-Br	61 ± 1	40 ± 2
AZA*	–	19 ± 2	17 ± 4

*Acetazolamide (**AZA**) was used as a standard inhibitor for both hCA I and II isoenzymes. Mean ± standard error from 3 different assays.

It can be seen from the results in Table 1 that all compounds in the series had a higher K_I_ compared to **AZA** towards hCA I. According to the Table 1, 4-fluoro substituted derivative compound **2** and non-substituted derivative compound **1**, had the lowest K_I_ values (21 ± 4 nM and 28 ± 6 nM, respectively) in the series towards hCA I. The 4-fluoro substituted derivative compound **2** had the most effective inhibitory activity with a similar K_I_ value (21 ± 4 nM) with **AZA** against hCA I.

According to the K_I_ values of the compounds against hCA II, the most active compounds in the series were the 4-ethoxy substituted derivative compound **4** and the 4-ethyl substituted derivative compound **6** with their lower K_I_ values than **AZA** (16 ± 6 nM and 16 ± 2 nM, respectively). On the other hand, the non-substituted derivative **1** and the 4-bromo substituted derivative **3** had similar K_I_ values, comparable to those of the reference drug, **AZA** (18 ± 5 nM and 19 ± 4 nM, respectively). When the K_I_ values towards hCA II in Table 1 are evaluated, it was observed that substitution at the *para* position of the phenyl ring leads to more effective inhibitors compared to the *ortho* or *meta* substitutions. Probably, this is due to the steric impairments in which the latter two substitution patterns participate within the costrained active site of the enzyme, as compared to the less sterically impaired *para* substitution, as observed for many other types of CAIs^32–41^.

## 4. Conclusions

We report a series of 1,3-diaryltriazene substituted metanilamide derivatives **1–12**, acting as CA inhibitors against the widely spread cytosolic isoforms hCA I and II. According to the inhibition results, the 4-fluoro substituted derivative compound **2** can be considered as a lead molecule due to its interesting inhibition profile against both hCA I and hCA II, making it of interest in systems where the activity of the two cytosolic isoforms is dysregulated.
